# Predictors of CD4 count changes over time among children who initiated highly active antiretroviral therapy in Ethiopia

**DOI:** 10.1186/s41182-020-00224-9

**Published:** 2020-05-22

**Authors:** Tilahun Yemanu Birhan, Lemma Derseh Gezie, Destaw Fetene Teshome, Malede Mequanent Sisay

**Affiliations:** Department of Epidemiology and Biostatistics, Institute of Public Health, College of Medicine & Health Sciences, University of Gondar, Gondar, Ethiopia

**Keywords:** Highly active antiretroviral therapy, Child, HIV, Modeling, Risk factors, Sub-Sahara Africa

## Abstract

**Introduction:**

Human immunodeficiency virus (HIV) infection results in a gradual depletion of immune function, particularly CD4 cells. The CD4 assessment plays a significant role in assessing treatment responses and clinical decision-making for patients on combination antiretroviral therapy (ART) in resource-limited settings. However, new data on CD4 count changes are scarce; the volatility of CD4 counts after initiation of ART over time remains largely uncharacterized. This study aimed to identify the predictors of CD4 changes over time among HIV-infected children who began ART in Amhara, Ethiopia.

**Methods:**

A retrospective follow-up study was performed. A total of 983 HIV-infected children who initiated ART in government hospitals in the Amhara region between 2010 and 2016 were included using a simple random sampling technique. Data were extracted using a structured checklist. An exploratory data analysis was carried out to explain individual and average profile plots. The linear mixed model was used to identify the CD4 change count predictors over time. Variables with *p* value < 0.05 were considered statistically significant in a multivariable linear mixed regression analysis.

**Results:**

The mean CD4 count of the participants was 465.1 cells/mm^3^ with an average CD4 count increase of 30.06 cells/mm^3^ over 6 months from baseline CD4 count and ART initiation. Childhood age (*β* = − 0.015; 95% Cl − 0.021, − 0.009), opportunistic infection at ART initiation (*β* = − 0.044, 95% CI − 0.085, − 0.004), hemoglobin level (*β* = 0.013; 95% CI 0.004, 0.022), and baseline WHO clinical stage II (*β* = − 0.046, 95% CI − 0.091, − 0.0003) were significant predictors of CD4 changes over time.

**Conclusions:**

The average CD4 count increase was sufficient in HIV patients who began combined antiretroviral therapy over time. The younger age of the infant, the higher baseline level of hemoglobin, the baseline WHO clinical stage II, and opportunistic infections led to changes in CD4 counts. As a result, timely diagnosis and treatment of opportunistic infections will reduce the risk of opportunistic infections.

## Introduction

Human immunodeficiency virus infection results in a gradual decline in immune function, especially in critical immune cells called CD4 [1]. Highly active antiretroviral therapy, the key component of the HIV treatment strategy, is intended to inhibit HIV replication [2], restore and/or sustain immune functions or CD4 cells [3], and enhance the quality of life of infected patients [4]. Evidence has shown that sufficient CD4 response in most patients with ART is considered when there is a rise in the range of 50–150 cells/mm^3^ per year with an accelerated response in the first 3 months of treatment before a steady-state level is reached [5]. Nevertheless, absolute CD4 counts can fluctuate within individuals, and some patients may not have an adequate CD4 count recovery due to reasons other than poor ART response. Previous studies indicated that ART initiation at an older age [6], baseline low CD4 count [6–9], and WHO clinical stages III and IV [10] were correlated with lower CD4 count recovery. Other observational studies have found that opportunistic infections [11, 12], presence of chronic diarrhea [13], low baseline hemoglobin level [13], poor adherence to drugs [14], poor functioning status [15], and low body mass index (BMI) [16, 17] at baseline were significantly allied with weak CD4 count shifts. As a result, children with low CD4 counts are at an elevated risk for adverse effects, such as growth retardation [18], drug resistance [19], and the onset of other opportunistic infectious diseases [20–23] and death [24].

Monitoring of CD4 counts of patients on ART is highly recommended for analysis of reactions to care and clinical decision-making in resource-limited settings [19, 25]. However, there is limited evidence of improvements in CD4 counts and related factors among children in HIV care and treatment programs in Ethiopia. The heterogeneity of CD4 cell counts after the initiation of ART over time remains largely uncharacterized, and most published works do not identify factors related to their heterogeneity with long-term use of ART in the country. The goal of this study was therefore to identify the predictors of CD4 cell count changes over time among HIV-infected children under 15 years of age who initiated ART in public hospitals in the Amhara region of Ethiopia.

## Methods

### Study design and period

A retrospective follow-up study was conducted among HIV-infected pediatric patients (< 15 years of age) who initiated ART from January 2010 to February 2016.

### Study area and population

The study was conducted in government hospitals in Amhara National Regional State. The region is the second most populated, with an estimated total population of 19,602,512, situated in the northwest of Ethiopia. The region has 19 public hospitals, 801 health centers, 3302 health institutions, and 1031 private health facilities (clinics and hospitals). All public hospitals offer ART services to adults and children of Amhara and neighboring areas. According to the Amhara Regional Health Office survey, more than 2112 pediatric HIV-infected patients were successfully enrolled in ART in these hospitals and clinics in 2018. The study population included HIV-positive children who began ART therapy in these public referral hospitals. However, children were removed from the study if they were under 6 months of age because the response to HIV treatment should be satisfactory after 6 months of follow-up.

### Sample size and sampling procedure

The sample size was determined using the following assumptions; all subjects measured at *m* = 8 time points (no dropout), constant within-subject correlation (*ρ* = 0.5, *σ*^2^ = 1), 90% power, a difference of 0.25 at the two-sided test, and 95% confidence interval (CI).

$$ {\displaystyle \begin{array}{l}n=\frac{4{\sigma}^2\left(1+\left(m-1\right)\rho \right)\left({Z}_{\frac{\alpha }{2}}+{Z}_{1-\beta}\right)2}{md^2}\\ {}n=\frac{4\left(1+(7)0.5\right)\left(1.96+1.645\right)2}{8\left({0.25}^2\right)}=467.86\approx 468\end{array}} $$

Given the effect size of two and the incomplete rate of 5%, the total sample (983) was included in the analysis. The research was performed in six out of 19 public hospitals, chosen based on their substantial proportion of patients served. The sample was then allocated proportionately to the selected hospitals, namely the University of Gondar Specialized, Debre Markos, Debre Birhan, Dessie referral, Felege Hiwot specialized, and Debre Tabor general hospitals for the sake of representativeness. Using the pediatric ART registration book, we have created a sampling frame for each hospital using the list of all children on ART and aged 6 months to 15 years.

### Data collection tools and procedures

Pre-tested standardized data collection checklist built based on HIV/ART intake forms to retrieve routinely collected data from pediatric HIV patients who started ART in selected hospitals. The outcome variable is the CD4 count for selected participants, which is measured repeatedly over time for each person. These are repeated observations on individuals allowing a direct analysis of the transition from the baseline in which a single subject appears to be interrelated. Present literature guides the choice of explanatory variables. Variables have been grouped into the following categories: sociodemographic and clinical variables reported at ART entry and follow-up by ART clinics including sex, age, baseline CD4 count, baseline weight, regimen type, WHO stage, functional status, baseline hemoglobin level, disclosure, mother ARV status, and time in months. Basic demographic and clinical data were collected from medical records and time-dependent covariates such as CD4 count (mm^3^), viral load, and weight (kg) were assessed almost every 6 months by ART nurses from each participant. Six nurses working at the ART clinic were hired as data collectors and extracted the data after they had been trained for 2 days. The data collection process was supervised by three supervisors. Data processing took place in private rooms. The collected data were reviewed regularly for completeness and corrected by the main investigator and the supervisors.

### Data processing and analysis

The data were entered in Epi Info 7 and exported to STATA14 for analysis. Descriptive figures, such as numbers, percentages, measures, standard deviations, and quartiles were used to describe children’s characteristics. Besides, we performed an exploratory review of the data to direct our modeling process. Logarithmic transformation was implemented to get rid of skewness in CD4 data and all analyses were performed using transformed result data.

Individual profile plots were constructed on the first 100 subjects to provide a rough image of how subjects evolved and to provide explanations for variation between and within subjects. Also, this study presented ideas on random effects, beginning with an overview. Average profile plots were constructed to explain the overall mean shift in CD4 count. From this study, indications were obtained as to the functional type of the transition, and the transition depended on certain characteristics of the covariates. Not all measurements were uniformly spaced around different subjects, as a result of which smoothing was applied using the LOWESS process.

With a continuous outcome variable, the linear mixed model was used to control the longitudinal nature of the data. First, the null model only/unconditional linear model containing the random effect was fitted; then the fixed intercept and slop were fitted. Next, a model containing fixed covariates was implemented to illustrate the variability of the CD4 cell count. The three structures (identity, interchangeable, and unstructured) were built to choose the covariance structure that better matches the data and thus reduces the probability of model misspecification. Finally, the one with the lowest Akaike information criteria value was chosen.

To assess the factors associated with changes in CD4 cell count, a univariate analysis of the linear mixed model was tested for each baseline factor, and those considered to be significant (*p* value < 0.2) were chosen for the multivariable linear mixed regression model. The linear mixed model was favored over the generalizing estimated equation because we were interested in the subject-specific effect rather than the average population effect. Besides, the maximum likelihood parameter estimation approach was used.

## Results

### Demographic characteristics of participants

Out of a total of 983 HIV-infected children chosen for the study, 951 (96.74%) cases were examined with complete baseline data. The mean age of baseline participants was 6.5 (SD = 3.8) years with an average follow-up period of 21.31 (SD = 18.75) months. Of the total participants, 509 (53.5%) were male and 731 (76.6%) were primary caregivers for babies, mainly biological mothers (Table 1).

**Table 1:** Demographic characteristic of study participants in the Amhara region public hospitals (2010–2016)

Variable	Category	Number	%
Sex	Male	509	53.5
	Female	442	46.5
Primary caregiver	Parents	731	76.6
	Grandparents	93	9.8
	Relatives	68	7.2
	Others	59	6.1

### Clinical characteristics

Of the patients, 286 (29.9%) were at baseline in WHO clinical stage I; 323 (33.86%) had functional status and 691 (72.8%) had opportunistic infections (Table 2). The mean weight of the participants at baseline was 19.13 kg (SD = 9.50 kg).

**Table 2:** Clinical and medication characteristics of study participants in the Amhara region public hospitals (2010–2016)

Variable	Category	Number	%
Regimen type	d4T + 3TC + NVP	327	34.3
	d4T + 3TC + EFV	76	8.0
	AZT + 3TC + NVP	333	34.9
	AZT + 3TC + EFV	160	16.8
	Others	55	5.8
Functional status	Working	323	33.9
	Ambulatory	462	48.4
	Bedridden	166	17.5
BWHO stage	Stage I	286	29.9
	Stage II	309	32.4
	Stage III	287	30.1
	Stage IV	69	7.2
Disclosure	Yes	720	75.7
	No	231	24.3
Opportunistic infection	Yes	691	72.7
	No	260	27.3
MARV status	Yes	909	95.6
	No	42	4.4
Medication adherence	Poor	291	38.04
	Fair	117	15.29
	Good	357	46.67

*BWHO* baseline WHO stage, *MARV* mothers’ antiretroviral

### Baseline laboratory characteristics

The mean baseline hemoglobin levels and CD4 counts of the study participants were 12.4 mg/dl (SD = 4.15) and 465.1 (SD = 398.3) cells/mm^3^, respectively. Furthermore, the mean rise in CD4 counts for children was 30.06 cells/mm^3^ per 6 months or 60.12 cells/mm^3^ per year.

### CD4 count changes after initiating ART

#### Mean profile plot stratified by age

**Fig. 1:**
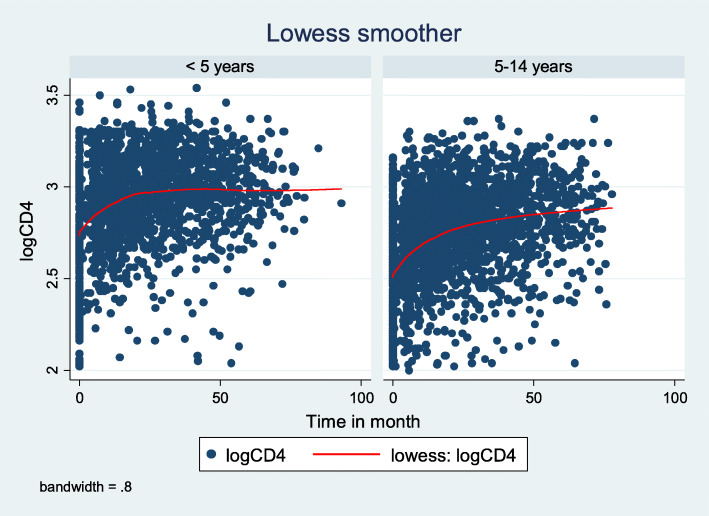
Smoothing mean profile plots for the logCD4 count after the initiation of ART among children in Ethiopia stratified by age from 2010 to 2016

In general, the average profiles of the first 100 patients showed signs of variation between and within subjects. The subjects had more variable CD4 values at the beginning and adjustments over time. This indicates that a theoretical linear mixed model with random intercepts and random slopes may be feasible starting points (Fig. 1).

#### Mean profile plot

**Fig. 2:**
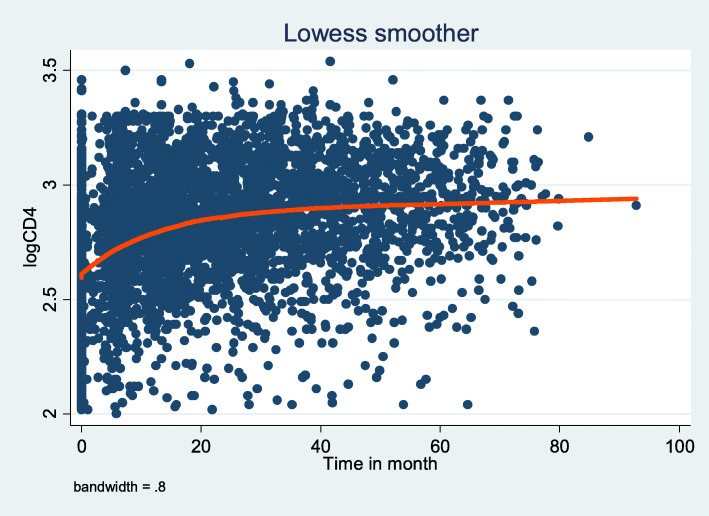
Smoothing mean profile plots for the logCD4 count after the initiation of ART among children in Ethiopia from 2010 to 2016

The average change in CD4 counts over time showed a dramatic rise in the first 20 months, followed by a modest increase of up to 40 months. In the end, it remained constant from the 40th to the last month. It is evident that the total mean CD4 count increased over time and remained constant over time (Fig. 2).

### Predictors of CD4 count changes over time

Throughout the modeling process, random intercept and random slopes were assumed to compensate for individual differences in CD4 cell count at baseline and over time, respectively. Also, random and fixed effects were calculated by comparing various covariance structures. In this case, the unstructured covariance structure offered the smallest information criterion and selected a random effect parameter model (Table S1 and S2). The values obtained from the study of the average logCD4 count of children at baseline were therefore 2.76, excluding covariate effects. The multivariable linear mixed model found covariates such as age, opportunistic infections, baseline WHO clinical stage II, and baseline hemoglobin levels were significantly correlated with CD4 count changes over time, while sex, HIV status, regimen type, adherence to medications, and maternal ARV status did not significantly affect CD4 cell count changes over time.

Age was closely correlated with CD4 cell count changes suggesting that the average logCD4 count decreased by 0.015 (*β* = − 0.015, 95% CI − 0.021, − 0.009) for every 1 year rise in children’s age. At baseline, the mean logCD4 count among children who had opportunistic infection was 0.044 (*β* = − 0.044; 95% CI − 0.085, − 0.004) times lower than the mean logCD4 count among children who had no opportunistic infection. Patients who began ART at WHO clinical stage II reported smaller increases in CD4 counts relative to those who began ART at the first stage. Moreover, the WHO stage negative coefficients indicate that their mean CD4 counts at baseline are significantly lower than the reference group (*p* < 0.001 for stage II vs. stage I). Baseline hemoglobin levels had a major positive impact on logCD4 count. In the case of a unit increase in hemoglobin, the logCD4 count increased by 0.013 (*β* = 0.013; 95% CI 0.004, 0.023).

Time has a positive significant impact on logCD4 count (*β* = 0.008; 95% CI 0.007, 0.009) suggesting that the average logCD4 count of a person rises by 0.0058 every 6 months are compatible with other covariates (Table 3). The standard deviation for the intercept was found to be 0.276. This indicates that there is a substantial difference in intercepts between research subjects and that the evolution of CD4 counts differs across children at baseline (Table S3).

**Table 3:** Multivariable linear mixed model analyses of factors associated with CD4 changes overtime among HIV-infected children-initiated ART Amhara region public hospitals (2010–2016)

Covariate	Estimate	95% CI	
Constant	2.494	2.328	2.660
Time	0.008	0.007	0.009
BMI	0.005	− 0.003	0.012
Age	− 0.015	− 0.021	− 0.009
Hemoglobin	0.013	0.004	0.022
BWHO stage			
Stage I	0		
Stage II	− 0.046	− 0.091	− 0.0003
Stage III	− 0.039	− 0.089	0.011
Stage IV	− 0.053	− 0.149	0.043
Opportunistic infection			
Yes	− 0.044	− 0.0845	− 0.0042
No	0		
Functional status			
Working	0		
Ambulatory	0.004	− 0.036	0.044
Bedridden	0.008	− 0.209	0.225
Disclosure			
Yes	− 0.087	− 0.129	0.046
No	0		
Mothers ARV status			
Yes	0.055	− 0.019	0.128
No	0		
Regimen type			
d4T + 3TC + NVP	0		
d4T + 3TC + EFV	− 0.143	− 0.217	0.068
AZT + 3TC + NVP	0.022	− 0.020	0.064
AZT + 3TC + EFV	0.070	− 0.017	0.123
TDF + 3TC + EFV	0.170	− 0.010	0.330
Others*	0.157	− 0.004	0.310

*TDF + 3TC + EFV, AZT + 3TC + LPV/r, ABC + 3TC + LPV/r

## Discussion

The goal of this study was to determine the rate of CD4 count change over time and to identify its predictors among HIV-infected children who initiated ART. The individual profile plot revealed the presence of variation in CD4 counts within and between individuals. The mean profile plot also indicated that, on average, logCD4 counts appeared to increase rapidly following initiation of antiretroviral therapy. In this analysis, the average CD4 count increment was 30.06 cells/mm^3^ per 6 months or 60.12 cells/mm^3^ per year.

This finding is consistent with the outcome of the Swiss HIV cohort study, which found that the correct CD4 response for most patients on therapy ranged from 50 to 150 cells/mm^3^ per year with an accelerated response in the first 3 months of treatment [5]. It may be due to the degree of suppression of viral replication, although it should be recognized that such plots are mean plots that may be different from individual plots, which may indicate that certain patients respond better than others. The study also attempted to classify the predictors of CD4 count changes over time. The age and CD4 count changes had negative associations, and the CD4 count log-odds decreased by 0.015 for a year at the age of the boy. This finding is consistent with those of studies conducted in northwest Ethiopia [15], India [26], EuroSIDA study of European countries [27], USA [28], and the Parirenyatwa Family Care Hospital [29]. It is well known that as age rises, thymic activity decreases and this will reduce CD4 counts as the thymus is the primary site of CD4 development [30]. Opportunistic infections had major negative effects on CD4 count changes over time, suggesting that children with opportunistic infections had lower CD4 counts. This is consistent with those of studies done in northwest Ethiopia [15], South Africa [31], and Korea [32]. This is because opportunistic infections improve HIV pathogenesis and further reinforce the value of prophylaxis [33].

Baseline WHO clinical stage II had a major negative impact on the CD4 count shift. As a result, the CD4 log-odds decreased by 0.0457 among HIV-infected children in stage II relative to a stage I patients. This result is consistent with the results of studies conducted in Ghana [34] and the Houston VA Special Medicine Clinic [35]. This has shown that children with more serious WHO clinical stages display a major decrease in CD4 absolute counts.

Hemoglobin level is also associated with an improved CD4 count over time. The finding is consistent with previous studies in South Africa [31] and Bomso Hospital, Kumasi, Ghana, [36] which indicated that a high level of hemoglobin is associated with increased CD4 counts, improved immune repair, and improved survival resulting in slower disease progression. The explanation is that a high level of hemoglobin affects the natural history of HIV by reducing the rate of progression of the disease [37].

The limitation of the analysis is that the causal relation cannot be precisely defined due to the retrospective nature of the results, which did not contain all the necessary variables such as viral load, other clinical parameters due to lack of reagents, or technical problems. Second, the impact of CD4 counts can fluctuate due to a lack of quality and availability of data. However, since a random effect model was used, the contribution of all the variables not calculated was pooled out of the total variation to obtain the modified effect of the independent variables considered.

## Conclusions

The average rate of CD4 count change was adequate in HIV patients who began combination antiretroviral therapy. The advanced age of the child, the higher baseline level of hemoglobin, the baseline WHO clinical stage II, and opportunistic infections contributed to changes in CD4 counts. As a result, early diagnosis and treatment of opportunistic infections reduce the risk of opportunistic infections. The pace of progress needs to be increased by continuing to expand universal access to ART, which can be scaled to have a population-level effect.

## Supplementary information

**Additional file 1: Table S1.** Selection of correlation structure for ART data set taken from the Amhara region from 2010-2016.

**Additional file 2: Table S2.** Random Effects Models with the associated values for the likelihood ratio test and p-value for ART data set taken from the Amhara region from 2010-2016.

**Additional file 3: Table S3.** Random parameter estimates for ART data set collected in the Amhara region from 2010-2016.

## Data Availability

The minimal data underlying all the findings in the manuscript will be available upon request.

## Abbreviations

3TC: Lamivudine
ABC: Abacavir
AIDS: Acquired immune deficiency syndrome
AIC: Akaike information criteria
ART: Antiretroviral therapy
ARV: Antiretroviral
AZT: Zidovudine
BIC: Bayesian information criteria
BMI: Body mass index
d4T: Stavudine
EFV: Efavirenz
ART: Highly active antiretroviral therapy
HIV: Human immunodeficiency virus
LMM: Linear mixed model
TDF: Tenofovir
WHO: World Health Organization
