# Supplementary material for: Predictors of CD4 count changes over time among children who initiated highly active antiretroviral therapy in Ethiopia
Source: Trop Med Health. 2020 May 22;48:37. doi: 10.1186/s41182-020-00224-9 (PMC7243309; doi:10.1186/s41182-020-00224-9)
Supplement: Supplementary file 1 — Additional file 1: Table S1. Selection of correlation structure for ART data set taken from the Amhara region from 2010-2016. [file 41182_2020_224_MOESM1_ESM.docx]

Table S1: Selection of correlation structure for ART data set taken from the Amhara region from 2010-2016

| Correlation structure | AIC | BIC |
| --- | --- | --- |
| Identity | 12987.52 | 13099.52 |
| Exchangeable | 12982.36 | 13083.17 |
| Un structured | 12854.43 | 12955.23 |
