# Supplementary material for: Predictors of CD4 count changes over time among children who initiated highly active antiretroviral therapy in Ethiopia
Source: Trop Med Health. 2020 May 22;48:37. doi: 10.1186/s41182-020-00224-9 (PMC7243309; doi:10.1186/s41182-020-00224-9)
Supplement: Supplementary file 2 — Additional file 2: Table S2. Random Effects Models with the associated values for the likelihood ratio test and p-value for ART data set taken from the Amhara region from 2010-2016. [file 41182_2020_224_MOESM2_ESM.docx]

Table S2: Random Effects Models with the associated values for the likelihood ratio test and p-value for ART data set taken from the Amhara region from 2010-2016

| Random effects | Likelihood-ratio test | p-value |
| --- | --- | --- |
| Model1: intercepts | 76.72 | < 0.001 |
| Model2: intercepts, time | 35.48 | < 0.001 |
