# Supplementary material for: Predictors of CD4 count changes over time among children who initiated highly active antiretroviral therapy in Ethiopia
Source: Trop Med Health. 2020 May 22;48:37. doi: 10.1186/s41182-020-00224-9 (PMC7243309; doi:10.1186/s41182-020-00224-9)
Supplement: Supplementary file 3 — Additional file 3: Table S3. Random parameter estimates for ART data set collected in the Amhara region from 2010-2016. [file 41182_2020_224_MOESM3_ESM.docx]

Table S3: Random parameter estimates for ART data set collected in the Amhara region from 2010-2016.

| Random intercept parameters | Estimate | Std error | 95% CI |
| --- | --- | --- | --- |
| Sd(b_oi_) | 0.276 | 0.008 | 0.189, 0.298 |
| sd(b_1i_) | 0.004 | 0.001 | 0.0003, 0.005 |
| corr(b_1i_  ,b_0i_) | 0.729 | 0.247 | -0.961, 0.107 |
| sd(є_ij_) | 0.163 | 0028 | 0.158 0.169 |
